# Atypical and inflexible visual encoding in autism spectrum disorder

**DOI:** 10.1371/journal.pbio.3001293

**Published:** 2021-06-08

**Authors:** Emily L. Isenstein, Woon Ju Park, Duje Tadin

**Affiliations:** 1 Department of Brain and Cognitive Sciences, University of Rochester, Rochester, New York, United States of America; 2 Department of Neuroscience, University of Rochester Medical Center, Rochester, New York, United States of America; 3 Center for Visual Science, University of Rochester, Rochester, New York, United States of America; 4 Department of Psychology, University of Washington, Seattle, Washington, United States of America; 5 Department of Ophthalmology, University of Rochester Medical Center, Rochester, New York, United States of America

## Abstract

Encoding, which involves translating sensory information into neural representations, is a critical first step in the sensory-perceptual pathway. This Primer explores the implications of a new study which uses a visual orientation task to reveal both lower encoding capacity and less flexible encoding adaptation in people with autism spectrum disorder.

## Teaser

Encoding, which involves translating sensory information into neural representations, is a critical first step in the sensory-perceptual pathway. Using a visual orientation task, a new study found both lower encoding capacity and less flexible adaptation in people with autism spectrum disorder.

Cursory understanding of autism spectrum disorder (ASD) links the condition mostly with social and communication difficulties as well as repetitive behaviors. However, differences in sensory perception are widely reported in people with ASD, particularly in the visual domain [1]. In recent years, there has been a steadily growing effort toward elucidating the root causes of these differences. The goal is not only to understand perceptual processing in ASD but also to consider how these differences in sensory processing might have cascading effects on cognitive and motor functions [1]. This effort faces a key obstacle: Perceptual processing is complex, involving multiple interconnected stages that utilize over half of the human cortex. This creates a challenging credit assignment problem, where it is difficult to link an observed atypicality in perceptual processing to a specific component of the perceptual pathway. The work by Noel and colleagues in this issue of *PLOS Biology* [2] takes an important step toward addressing this challenge.

**Fig 1:**
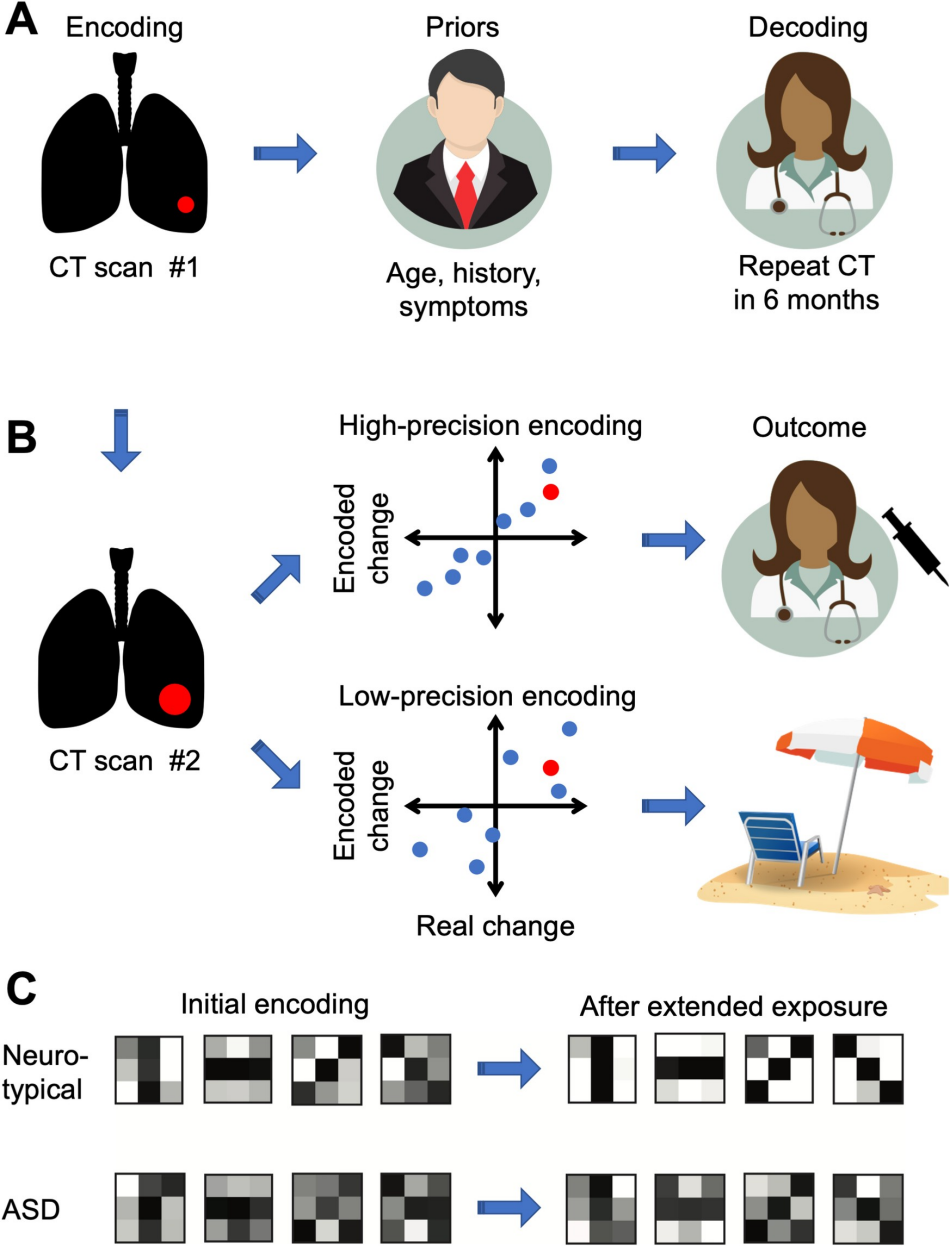
Perceptual encoding and decoding. (A) Medical imaging can be used as an analogy for perceptual encoding and decoding. Encoding is the mapping of sensory inputs onto neural representations, which, in our analogy, can be thought of a CT machine capturing an image of a lung mass. The interpretation of these images by a doctor illustrates perceptual decoding. Here, the doctor’s assessment is influenced by prior knowledge (e.g., patient’s background, age, and symptoms). Similarly, our perception is strongly influenced by our vast prior experience with our sensory environment. (B) In this analogy, the patient returns for a second CT. If the CT machine has high-precision encoding, then even a small increase in the measured size of the mass may suggest a significant change, and the doctor will order a biopsy. If the CT machine has low-precision encoding, then an equally small increase in the measured size of the mass may be within the margin of error, and the doctor may not order a biopsy. Similarly, high-precision perceptual encoding will lead to quick detection of changes in sensory statistics and lead to an appropriate adjustment in perceptual processing. Such changes in sensory statistics, however, may remain undetected when perceptual encoding is low in precision. (C) Noel and colleagues found that individuals with ASD have poor encoding precision of visual orientation (left), here illustrated as a noisier encoding of 4 orientations (vertical, horizontal, and two obliques; noisier encoding is depicted as less pronounced differences between dark orientation signals and the surrounding pixels). After extended exposure to an artificial statistical distribution of orientations, neurotypical individuals exhibited a significant improvement in encoding that matches the novel statistics (top right), in contrast to the ASD group, which showed little change (bottom right). ASD, autism spectrum disorder; CT, computerized tomography.

Broadly speaking, our perception of the world is a result of an “encoding–decoding” process. Encoding describes how low-level sensory representations of the world are generated by mapping sensory inputs onto noisy and resource-limited neural representations. This, however, is not the end of perception. Perceptual processing fundamentally depends on how these sensory representations are decoded based on various “rules,” including combining sensory measurements with prior knowledge (Fig 1A). The result is effective and flexible perceptual functioning that takes advantage of our vast perceptual knowledge. In ASD research, much of the recent work focused on alterations in the decoding stage as a way to explain sensory differences and how they translate to behavior in this population [3]. In contrast, Noel and colleagues used a combination of behavioral methods and computational modeling to isolate the early “encoding” stage of perceptual processing.

To study visual encoding, Noel and colleagues used a simple orientation estimation task. Here, participants briefly viewed an oriented stimulus and subsequently reported the perceived orientation. This task reveals a characteristic pattern of repulsive biases away from cardinal orientations [4]. For example, a stimulus tilted 15° away from vertical is perceived as having a 25° tilt. This pattern of bias has been linked to “efficient encoding” of common orientations in the natural environment where horizontals and verticals are overrepresented [4,5]. In the first block of the experiment, both neurotypical and ASD groups exhibited this characteristic pattern of bias. However, individuals with ASD had 60% higher variance in their orientation estimates. This result is consistent with previous work showing noisier orientation perception in ASD [6]. The key advance of the present study is the use of bias and variance data to compute the “Fisher information”—how much information participants’ responses carry about the actual stimulus orientation. Here, the authors relied on a well-known property of an estimator in statistics—the “Cramer–Rao bound”—which tells us the best performance we can ever expect from an unbiased estimator. Simply stated, poor stimulus encoding will result in highly variable stimulus estimates. When the estimator is biased, as is the case for when we perceive orientations, this bound sets a relationship between Fisher information and the two things an experimenter can measure: bias and variance. The key insight is that this relationship holds regardless of the differences in decoding scheme, allowing the experimenter to isolate the encoding capacity.

As foreshowed by increased response variance, individuals with ASD had lower baseline encoding capacity than neurotypical controls (Fig 1C, left). Fisher information for both groups peaked at cardinal orientations. However, the neurotypical group had about 30% higher encoding capacity overall. This finding is consistent with noisier, more variable sensory processing in ASD reported in both neuroimaging [7] and behavioral work [6] (but see [8]).

The second key finding provides insights into how individuals with ASD adapt to changes in stimulus statistics. Under the efficient coding hypothesis, visual neurons are optimized to maximize the information they carry about the natural environment [5]. Thus, in the face of changes in visual input statistics, the hypothesis predicts that the visual system will reallocate encoding resources to adapt to the novel statistics. The present study gave participants increasing experience with an artificial environment where, in contrast to natural statistics, the distribution of orientations was uniform. As expected, in the neurotypical group, participants’ encoding capacity increased over the course of the experiment and was reallocated to better match the uniform distribution of orientations used in the experiment. Individuals with ASD, however, did not exhibit a change in the encoding strategy (Fig 1C, right). Consequently, by the end of the experiment, the group difference in the encoding capacity widened from 30% to 50%.

What are the implications of this pair of findings? Reduced perceptual encoding might lead to less reliable and less predictable sensory representations of the environment. This may affect how individuals with ASD interact with the sensory world [1,3,6]. The finding of inflexible adaptation to a new sensory environment further suggests that people with ASD may need additional time to acclimate to new circumstances, a concept central to the behavioral inflexibility and adherence to routine commonly reported in this group. These results align with other work that has identified challenges of updating expectations based on new circumstances in ASD [9,10]. We do stress that these interpretations of the possible cascading effects of atypical sensory encoding are speculative. Furthermore, while the empirical results by Noel and colleagues are rather convincing with large differences between the groups, they are not without limitations. The key outstanding question is the degree to which these results generalize to overall perceptual functioning—this subject will require follow-up research. Data on perceptual function in ASD are rather heterogenous and include both examples of diminished and enhanced perception [1]. For example, one theory postulates highly precise but inflexible encoding in ASD [11] that seemingly contradicts the current results. There are also technical issues to consider. In Noel and colleagues’ work, the stimulus disappeared before participants made their responses. Thus, processes that may not be related to sensory encoding, such as perceptual memory, may play a role in the reported results.

An additional obstacle in finding root causes of differences in behavior is to distinguish the atypicalities that are fundamental to ASD and those that are compensatory. Noel and colleagues offer a good example here. The finding of inflexible adaptation to stimulus statistics might seem fundamental, but the authors show that this behavior is correlated with the initial differences in the encoding capacity. One explanation of this result is that someone with poor sensory encoding would have more trouble detecting a change in the sensory environment and consequently delay the use of appropriate adaptation mechanisms (Fig 1B). We suspect that analogous explanations may exist for many symptoms that are reported in ASD, where the observed differences are not a reflection of aberrant brain function, but the brain’s adaptive response to something else being atypical. A better understanding of this distinction will likely be critical for evaluating the impacts of atypical and inflexible visual encoding on clinical behaviors in ASD as well as for identifying more precise targets for interventions.
